# Untying the Gordian knot of plastid phylogenomic conflict: A case from ferns

**DOI:** 10.3389/fpls.2022.918155

**Published:** 2022-11-24

**Authors:** Ting Wang, Ting-Zhang Li, Si-Si Chen, Tuo Yang, Jiang-Ping Shu, Yu-Nong Mu, Kang-Lin Wang, Jian-Bing Chen, Jian-Ying Xiang, Yue-Hong Yan

**Affiliations:** ^1^ Key Laboratory of National Forestry and Grassland Administration for Orchid Conservation and Utilization, The Orchid Conservation and Research Center of Shenzhen, Shenzhen, China; ^2^ Yunnan Academy of Biodiversity, Southwest Forestry University, Kunming, China; ^3^ College of Forestry and Landscape Architecture, South China Agricultural University, Guangzhou, China; ^4^ Eastern China Conservation Centre for Wild Endangered Plant Resources, Shanghai Chenshan Botanical Garden, Shanghai, China; ^5^ Green Development Institute, Southwest Forestry University, Kunming, China

**Keywords:** phylogeny, gene tree conflict, plastome, slowly evolving genes, Pteridineae

## Abstract

Phylogenomic studies based on plastid genome have resolved recalcitrant relationships among various plants, yet the phylogeny of Dennstaedtiaceae at the level of family and genera remains unresolved due to conflicting plastid genes, limited molecular data and incomplete taxon sampling of previous studies. The present study generated 30 new plastid genomes of Dennstaedtiaceae (9 genera, 29 species), which were combined with 42 publicly available plastid genomes (including 24 families, 27 genera, 42 species) to explore the evolution of Dennstaedtiaceae. In order to minimize the impact of systematic errors on the resolution of phylogenetic inference, we applied six strategies to generate 30 datasets based on CDS, intergenic spacers, and whole plastome, and two tree inference methods (maximum-likelihood, ML; and multispecies coalescent, MSC) to comprehensively analyze the plastome-scale data. Besides, the phylogenetic signal among all loci was quantified for controversial nodes using ML framework, and different topologies hypotheses among all datasets were tested. The species trees based on different datasets and methods revealed obvious conflicts at the base of the polypody ferns. The topology of the “CDS-codon-align-rm3” (CDS with the removal of the third codon) matrix was selected as the primary reference or summary tree. The final phylogenetic tree supported Dennstaedtiaceae as the sister group to eupolypods, and *Dennstaedtioideae* was divided into four clades with full support. This robust reconstructed phylogenetic backbone establishes a framework for future studies on Dennstaedtiaceae classification, evolution and diversification. The present study suggests considering plastid phylogenomic conflict when using plastid genomes. From our results, reducing saturated genes or sites can effectively mitigate tree conflicts for distantly related taxa. Moreover, phylogenetic trees based on amino acid sequences can be used as a comparison to verify the confidence of nucleotide-based trees.

## Introduction

**Figure 1:**
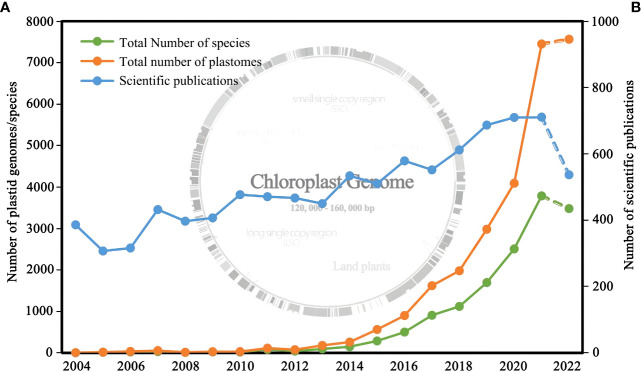
**(A)** Number of plastid genomes of land plants released and the corresponding number of species from 2006 to 2022; **(B)** Number of research articles published from 2006 to 2022. Genomic data were obtained from NCBI (filter criteria: land plant, 120,000-160,000 bp, chloroplast), and the publication data were obtained from Web of Science (filter criteria: plant, phylogeny, chloroplast).

With the development of next-generation sequencing technology and a decrease in cost, plastid (chloroplast) genomes (plastomes) have become more accessible in recent years (Edwards and Batley, 2010; Barrett et al., 2013; Liao et al., 2020). Besides, due to their highly conserved structure and relatively low nucleotide substitution rates compared with the nuclear genome (Daniell et al., 2016), plastid genomes have been widely employed to resolve evolutionary relationships among lineages of plant (Palmer, 1985; Roure and Philippe, 2011; Yang et al., 2019; **Figure 1**), such as Alismatales (Ross et al., 2016) and Ptilidiales (Yu et al., 2020). These studies greatly advanced and honed our understanding of plant evolutionary relationships; however, several problems, such as ancient adaptive radiations events (Barrett et al., 2013), hybridization and intragenomic conflict, remain unsolved due to relatively small sets of plastid genes used in the analysis. Therefore, the reliability of plastid genes or an entire plastome is ultimately determined based on the extent to which they reflect the “true” evolutionary relationships of the lineages (Doyle, 1992; Walker et al., 2019).

Numerous studies have shown that phylogenomic conflict (gene trees disagreement about species tree resolution; Caroline et al., 2021) is a nearly ubiquitous feature of nuclear or nuclear-plastid phylogenomic (Rokas et al., 2003; Smith et al., 2015; Liu et al., 2016; Zhang et al., 2019), which is attributed to biological (e.g., hybridization, duplication, incomplete lineage sorting and horizontal gene transfer) and non-biological factors (e.g., systematic error, uninformative loci, outlier genes and gene saturation; Maddison and Wiens, 1997; Galtier and Daubin, 2008; Vargas et al., 2017; Walker et al., 2017). Although nuclear or nuclear-plastid conflicts in phylogenetic analysis have been thoroughly investigated (Sun et al., 2015; Zhang et al., 2019; Liu et al., 2020; Stull et al., 2020), conflicts within the plastome are still poorly explored (Gonçalves et al., 2019; Walker et al., 2019; Xiao et al., 2020; Zhang et al., 2020a), possibly because the plastome is typically uniparentally inherited (Birky, 1995; Mogensen, 1996; Vargas et al., 2017). Moreover, stochastic and systematic errors or misspecifications of the evolutionary model (Walker et al., 2019; Daniell et al., 2021) also cause internal conflicts. Therefore, many researchers are used to simply combining various plastid sequences to amplify the phylogenetic signal (Gadagkar et al., 2005; Kumar et al., 2012) and infer evolutionary relationships among recalcitrant lineages, such as Polypodiaceae (Du et al., 2021; Wei et al., 2021) and Poales (Givnish et al., 2010). However, the tree topology based on this approach may be incorrect, even with high support values (Walker et al., 2017; Lu et al., 2018; Gonçalves et al., 2019; Walker et al., 2019).

Recent studies on plastid genomes have identified biparental inheritance in some angiosperm and fern species, such as *Passiflora sp*. (Hansen et al., 2007), *Silene vulgaris* (McCauley et al., 2007), *Pereskia aculeata* (Zhang et al., 2003), *Equisetum arvense* (Renzaglia et al., 2002; Crosby and Smith, 2012) and *Selaginella moellendorffii* (Renzaglia et al., 1999; Crosby and Smith, 2012), indicating the possibility of chimeric plastomes and heteroplasmy. Besides, the plastid genome could share genes with the nuclear and mitochondrial genomes (Hansen et al., 2007; Rice et al., 2013; Smith, 2014); however, this has rarely been found in plants (Smith, 2014). These patterns could result in gene tree conflicts in plastome-inferred phylogenies mentioned above in angiosperms, such as commelinids, rosids, and Fabaceae (Barrett et al., 2013; Gonçalves et al., 2019; Walker et al., 2019; Zhang et al., 2020a). Although there were reports of plastid conflicts, few methods have been identified to solve these conflicts and obtain a relatively stable and reliable phylogenetic tree.

**Figure 2:**
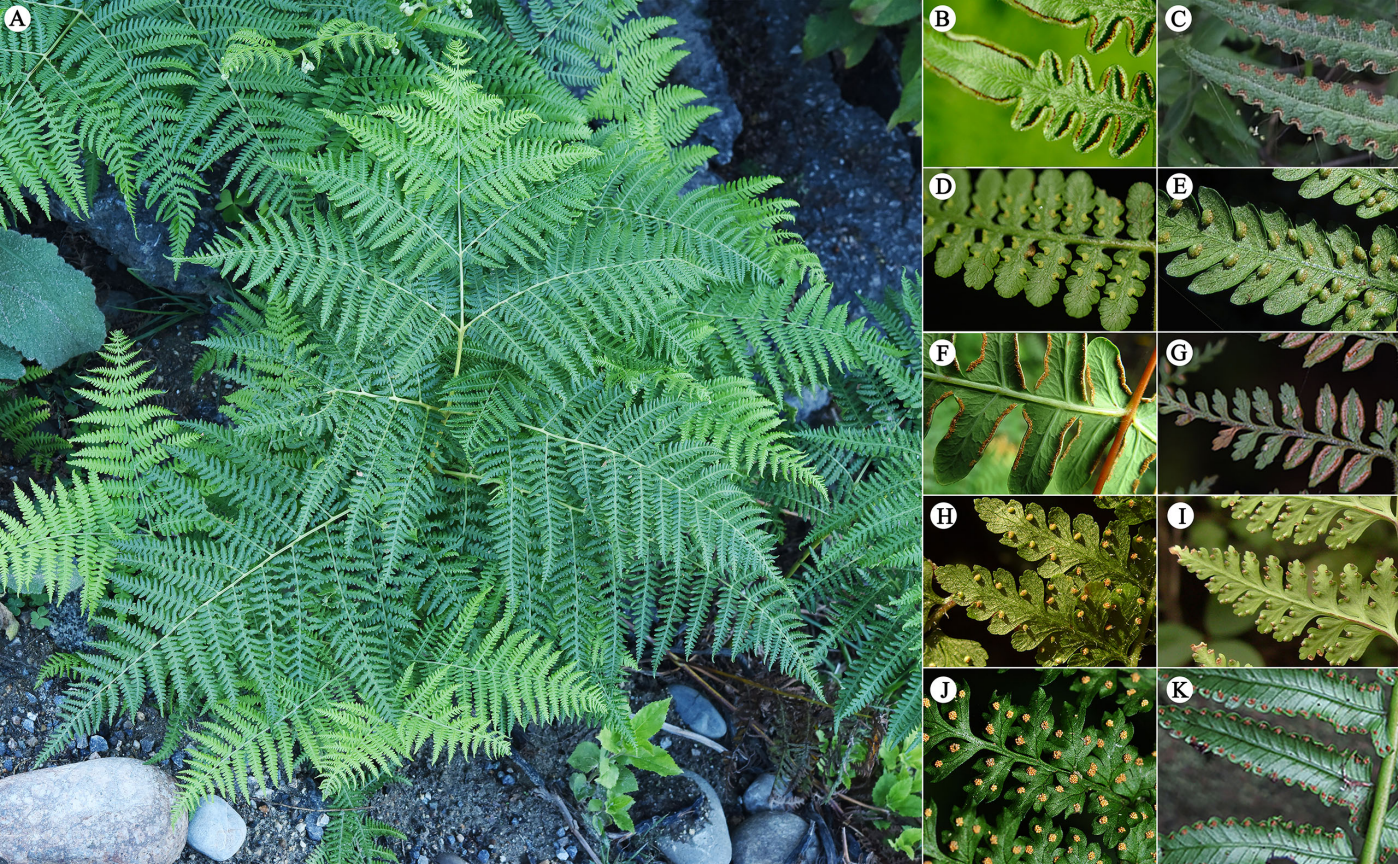
Morphological characteristics of Dennstaedtiaceae. **(A–G)** Hypolepidoid clade; **(A, B)** *Pteridium aquilinum*; **(C)** *Blotiella* sp.; **(D)** *Hiya brooksiae*; **(E)** *Hypolepis tenuifolia*; **(F)** *Histiopteris incisa*; **(G)** *Paesia radula*; **(H, I)** Dennstaedtioid clade; **(H)** *Microlepia hancei*; **(I)** *Dennstaedtia scabra* var. *glabrescens*; **(J-K)**. Monachosoroideae; **(J)** *Monachosorum henryi*; **(K)** *Monachosorum maximowiczii*.

Dennstaedtiaceae Lotsy is a medium-sized family in ferns, which contains 11(–15) genera and 170–300 species (**Figure 2**), widespread in the tropical and temperate regions (Yan et al., 2013). Edge-colonizing habit, chromosomal aneuploidy, polyploidy, and hybridization are standard features of most species of Dennstaedtiaceae (Schwartsburd et al., 2020). Extensive phylogenetic analysis recovered Dennstaedtiaceae as a monophyletic family comprising three subfamilies, Dennstaedtioideae C.Chr. nom. nud. (Dennstaedtioid clade), Hypolepidoideae Lovis nom. nud. (Hypolepidoid clade) and Monachosoroideae Crabbe, Jermy & Mickel (Pryer et al., 2004; Schuettpelz and Pryer, 2007; Lu et al., 2015; Perrie et al., 2015; Rothfels et al., 2015; Liu, 2016; Ivan and Schwartsburd, 2017; Shang et al., 2018; Schwartsburd et al., 2020; Du et al., 2021). The Dennstaedtioideae (Dennstaedtioid clade) comprises *Microlepia* C. Presl, *Oenotrichia* Copel. *Leptolepia* Prantl and a polyphyletic *Dennstaedtia* Bernh. (PPGI, 2016; Shang et al., 2018; Schwartsburd et al., 2020); the Hypolepidoideae (Hypolepidoid clade) comprises *Blotiella* R.M. Tryon, *Histiopteris* (J.Agardh) J.Sm., *Hiya* H. Shang, *Paesia* A.St.-Hil., *Hypolepis* Bernh. and *Pteridium* Gled. ex Scop.; the Monachosoroideae includes only *Monachosorum* Kunze (Perrie et al., 2015; Liu, 2016; Shang et al., 2018; Schwartsburd et al., 2020). Phylogenetic relationships within the genera of Dennstaedtiaceae has become clearer since the development of molecular systematics, except for the *Dennstaedtia* (Schwartsburd et al., 2020). In addition, the closely relatives of Dennstaedtiaceae are Pteridineae and eupolypods, both of which are the most diverse groups of ferns.

**Figure 3:**
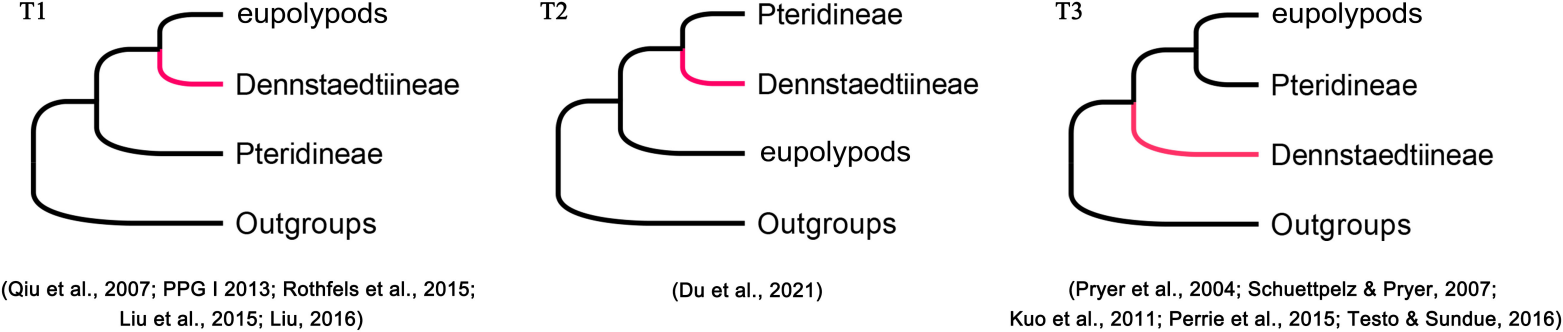
Phylogenetic position of Dennstaedtiineae among the polypod ferns suggested in the previous studies.

Phylogenetic position of Dennstaedtiaceae among the polypod ferns has not been resolved in the past decades (Lu et al., 2015; Perrie et al., 2015; Rothfels et al., 2015; Liu, 2016; Shang et al., 2018; Shen et al., 2018; Liu et al., 2020; Du et al., 2021) due to different studies have inferred different topologies. Based on plastid data, three topologies (T1, T2, and T3; **Figure 3**) have been recovered among Dennstaedtiaceae, Pteridineae, and eupolypods: (T1) Dennstaedtiaceae as sister to the eupolypods (Qiu et al., 2007; Lu et al., 2015; Liu, 2016; PPGI, 2016); (T2) Dennstaedtiaceae as sister to Pteridineae (Du et al., 2021); (T3) Dennstaedtiaceae as sister to the clade comprising Pteridineae and the eupolypods (Pryer et al., 2004; Schuettpelz and Pryer, 2007; Kuo et al., 2011; Perrie et al., 2015; Testo and Sundue, 2016). Nuclear data consistently supported topology T1 (Rothfels et al., 2015; Qi et al., 2018; Shen et al., 2018). Fewer informative loci, incomplete sampling, stochastic error (Walker et al., 2019; Du et al., 2021) or gene tree conflict (Gonçalves et al., 2019; Walker et al., 2019; Zhang et al., 2020a), may have led to the differences in topologies among the previous studies. To understand these discrepancies, the conflicting phylogenetic signals in polypod ferns need to be further analyzed.

Using an extensive sampling of newly generated plastomes and data available on online repositories, our study aims to resolve the most problematic nodes in the phylogeny of Dennstaedtiaceae, while exploring the distribution of phylogenetic signal and conflict across plastome-inferred phylogenies. The phylogeny was first inferred using plastomes of 72 species from 47 genera and 25 families, representing the major lineages within Dennstaedtiaceae, Pteridineae, and the eupolypods. Multiple strategies were adopted to minimize the systematic and inference errors, and a maximum likelihood framework was used to quantify the distribution of phylogenetic signal among genes for the controversial nodes and to test phylogenetic hypotheses. Dennstaedtiaceae represents an excellent system to explore the extent of conflict and impact on plastid phylogenomics, a topic that has been rigorously examined in plants only recently (Walker et al., 2019; Zhang et al., 2020a). The study’s findings will improve the understanding of the evolution of polypod ferns and provide a model for the phylogenomic analysis of related taxa (family level or above) based on plastomes.

## Materials and methods

### Taxon sampling and sequencing

In this study, 30 new Dennstaedtiaceae plastomes belonging to 29 species and 9 genera were sequenced. Combined with publicly available complete plastome data in National Center for Biotechnology Information (NCBI; https://www.ncbi.nlm.nih.gov/), plastomes from 72 species of 47 genera and 25 families were analyzed. Detailed information on the studied taxa, arranged according to the PPG I (PPGI, 2016) classification, is provided in **Supplementary Table S1**.

Total DNA was extracted from fresh, young leaves using Plant Genomic DNA Kit (Tiangen, Beijing, China), following the manufacturer’s protocol. DNA degradation and contamination were monitored on 1% agarose gels. DNA purity was determined with the NanoPhotometer^®^ spectrophotometer (Implen, CA, USA), and DNA concentration was measured using the Qubit^®^ DNA Assay Kit in a Qubit^®^ 2.0 Fluorometer (Life Technologies, CA, USA). The qualified DNA were fragmented by Covaris M220 Focused-ultrasonicator (Covaris, MA) instrument. The fragmented DNA was repaired at the end, followed by the addition of the sequencing adapter, and then the ~400 bp fragments of the genome were enriched through magnetic beads adsorption and amplified by PCR to form sequencing library. The libraries that passed the quality inspection were sequenced using the Illumina HiSeq 4000 platform according to the manufacturer’s instructions, and 150 bp paired-end reads were generated.

After the Illumina HiSeq 4000 sequencing data (Raw Data) was finished, the software Fastp 0.19.6 was used to control the quality of the Raw Data and filter the low-quality data to obtain high-quality Clean Data. The specific operations are as follows: 1) Remove Adapter sequence in Reads; 2) The bases whose sequencing quality value at the 5’ end was lower than 20 or identified as N were cut out; 3) The bases whose sequencing quality value at the 3’ end was lower than 3 or identified as N were cut out; 4) Take 4 bases as Window, and cut out the bases in Window with average mass value less than 20; 5) Reads containing 10% of N were removed; 6) More than 40% Reads with base quality values below 15 were cut out; 7) Reads with length less than 30 bp after removing Adapter and quality pruning were discarded.

### Plastid genome assembly, annotation and comparison

The paired-end reads of clean data were filtered and assembled into contigs using GetOrganelle pipeline (https://github.com/Kinggerm/GetOrganelle) with the parameters set as R (Maximum extension rounds) =15 and k (kmers) = 75, 85, 95, 105. The assembled plastomes were visually inspected and edited using Bandage (Wick et al., 2015), then a complete or nearly-complete circular plastome was generated for each sample. The annotation of plastomes was performed using PGA (Plastid Genome Annotator; Qu et al., 2019) with the reference plastome of *Histiopteris incisa* (MH319942), and then visually inspected and edited by hand where necessary in Geneious v11.1.5 (Kearse et al., 2012). The tRNA genes were also annotated in Geneious v11.1.5 using the reference genome of *H. incisa* with parameters set as sequence similarities more than 80%. Finally, 30 high-quality, complete plastid genome sequences were obtained. OrganellarGenomeDRAW (OGDRAW) v1.3.1 (https://chlorobox.mpimp-golm.mpg.de/OGDraw.html) was used to visualize the structural features of the plastomes of 31 species (Greiner et al., 2019).

In order to understand the sequencing and assembly quality, we aligned the clean data to the assembled plastid genome using the “Map of Reference” function of Geneious v11.1.5, and then obtained a visual coverage map of the plastid genome sequencing. According to the coverage map, the median read depth of each plastome was obtained.

### Sequence alignment and cleanup

**Figure 4:**
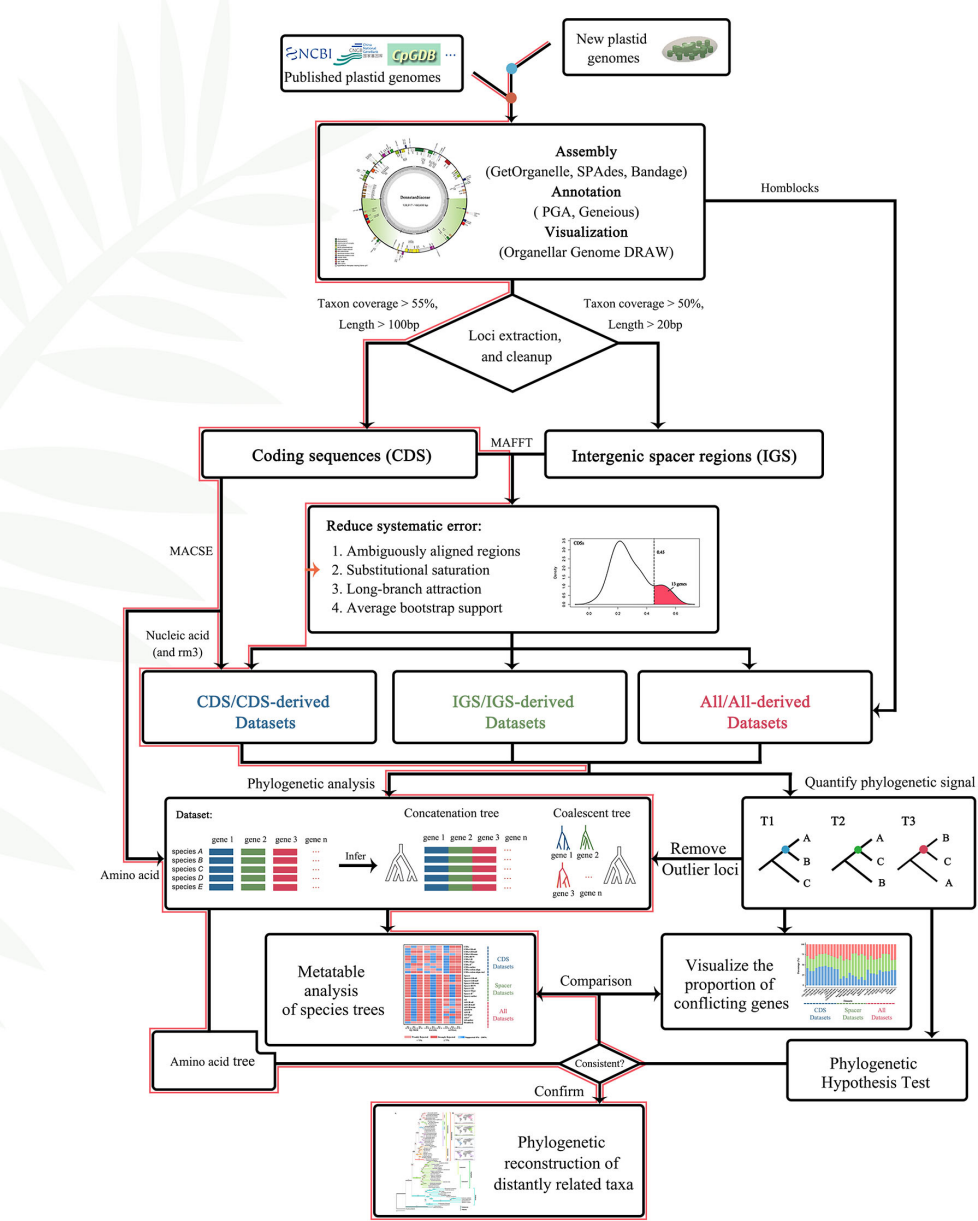
Flowchart of gene selection and phylogeny construction. The red lines represented the path to the most supported topologies in this article.

Furthermore, the “get_annotated_regions_from_gb.py” (https://github.com/Kinggerm/PersonalUtilities/) script was used to automatically extract all CDS (coding regions) and intergenic spacer regions from a list of annotated files in GenBank-format and manually correct the results. The CDS and intergenic spacer regions were individually aligned using the L-INS-i method of MAFFT v.7.475 (Katoh et al., 2002). Further, loci covering shorter than 55% of the species, and loci of CDS shorter than 100 bp or intergenic spacer regions less than 50 bp were removed to minimize the use of loci with limited information or present in relatively few species. Finally, 166 loci, including 81 CDS and 85 intergenic spacer regions, were obtained from 72 plastomes for downstream analysis (**Figure 4**).

### Datasets generation

The script “concatenate_fasta.py” (https://github.com/Kinggerm/PersonalUtilities/) was used to concatenate the alignments of each locus and create three basic datasets, the CDS, the IGS (intergenic spacer regions), and the All (the concatenated CDS and intergenic spacer regions) datasets. Furthermore, four strategies were applied to reduce the systematic error in the three basic datasets. The first strategy excluded ambiguously aligned regions using Gblocks v0.91b (Castresana, 2000) with relaxed, default and strict parameters (“Allowed Gap Positions” = “With All/Half/None”), generating the “CDS-GB-all”, “CDS-GB-half”, “CDS-GB-none”, “IGS-GB-all”, “IGS-GB-half”, “IGS-GB-none”, “All-GB-all”, “All-GB-half” and “All-GB-none” datasets. The second and third strategies identified and excluded loci with high levels of excessive substitutional saturation (slope and *R^2^* values) and evolutionary distance (long-branch score) using TreSpEx v.1.1 (Struck, 2014). Then, density plots of long-branch score, slope and *R^2^* values were generated with R v.3.2.2 (Pilson and Decker, 2002). The distribution of the long-branch scores of CDS and IGS loci showed a small shoulder at 0.45 and 0.90, respectively (**Figures S1A, D**), corresponding to the removal of 13 CDS loci and nine IGS loci from the CDS/IGS/All datasets to form “CDS-LB”, “IGS-LB”, and “All-LB” datasets. The 22 CDS loci (small shoulder at 0.50, the same below) and 18 IGS loci (0.344) located on the left “hump” of the *R^2^* distribution (**Figures S1B, E**) were trimmed from CDS/IGS/All datasets to generate the “CDS-*R^2^*”, “IGS-*R^2^*”, and “All-*R^2^*” datasets. Then, nine CDS loci (0.104) and 73 IGS loci (0.30; **Figures S1C, F**) located on the left “hump” of the slope distribution (**Figures S1B, C**) were removed from the CDS/IGS/All datasets to generate the “CDS-slope”, “IGS-slope”, and “All-slope” datasets. The fourth strategy used TreSpEx v.1.1 (Struck, 2014) to calculate the average bootstrap support (BS) of all nodes in the maximum likelihood trees generated from each of the 166 loci (**Table S2**) and then removing loci with less than 75% ultrafast bootstrap (UFBoot) support, generating the “CDS-BS75”, “IGS-BS75”, and “All-BS75” datasets. The loci excluded from each dataset are listed in **Supplementary Table S2**.

Furthermore, in order to compare the effects of different alignment methods, we also used “codon-aligned” and “homologous block searching” sequence alignment methods implemented in MACSE 0.9b (Ranwez et al., 2011) and Homblocks (Bi et al., 2018). Homblocks can automatically recognize locally collinear blocks and excavate core conserved fragment (protein coding genes, conserved non-coding regions, and rRNA genes) among plastid genomes (Bi et al., 2018), which produced the “All-Homblock” datasets from the All dataset. At the same time, we imported the “mauve.out” output file of Homblocks in Mauve (Darling et al., 2004) and visualized synteny blocks of 72 plastomes. Since only the CDS region of plastid genomes can be used for codon alignment, the “codon-aligned” strategy produced “CDS-codon-align” datasets from the CDS dataset. Subsequently, a new custom script "remove_third_codon.py" (https://github.com/TingWang-93/ferns) was developed and used to remove the third-codon positions of the “CDS-codon-align” dataset to form “CDS-codon-align-rm3” datasets. Numerous studies have shown a significantly higher substitution rate at the third-codon position when compared with the other two codon positions (Bofkin and Goldman, 2007; Mordecai et al., 2016; Katz, 2020), which may cause site saturation and mislead phylogenetic reconstructions (Breinholt and Kawahara, 2013; Struck, 2014).

For gene sequences that encode proteins, phylogenetic analysis can be performed based on either the nucleic acid or the amino acid sequences (Gupta, 1998). The analysis based on nucleic acid sequences, with three times as many characters, would seem to be more informative than amino acid sequences. While this is true in principle, for phylogenetic analysis involving distantly related taxa (family level or above), the increased information content in nucleic acid may be an illusion and, in most cases, a major liability (Gupta, 1998). Further, to confirm the accuracy of the final topologies, we translated the CDS dataset of 81 codon-alignment into amino acid for downstream phylogenetic analysis (**Figure 4**).

### Phylogenetic analyses

The maximum-likelihood (ML) and multispecies coalescent (MSC) methods were used to infer species and gene trees for both nucleic acids and amino acids datasets. For phylogenetic inference using the maximum-likelihood (ML) approach, we used two different heuristic search algorithms to test the deviation between the softwares. First, IQ-TREE v2.0.3 (Nguyen et al., 2015) was run with the –TEST and –AICc, and tree search options, using 1,000 ultrafast bootstrap replicates (Chernomor et al., 2014; Kalyaanamoorthy et al., 2017) with the best-fit model of evolution selected by ModerlFinder (Kalyaanamoorthy et al., 2017). Second, RAxML v8.2.12 (Alexandros, 2014) was run under the GTR + I + G substitution models. The support for the nodes in the phylogeny inferred with RAxML was assessed through rapid bootstrap (RBS) analysis with 500 pseudo-replicates. For phylogenetic inference using the MSC method, gene trees for each of datasets were inferred in IQ-TREE using the best-fit substitution model (determined by ModelFinder), followed by 1,000 independent likelihood searches from a random starting tree. To avoid arbitrary topologies detrimentally influencing the species tree, branches arising from nodes with less than 10% UFBS support value were collapsed on each tree (Zhang et al., 2017), and then used as input for ASTRAL-II v4.11.1 (Siavash and Tandy, 2015) with local posterior probability (LPP).

### Quantification of phylogenetic signals of alternative tree topologies

**Figure 5:**
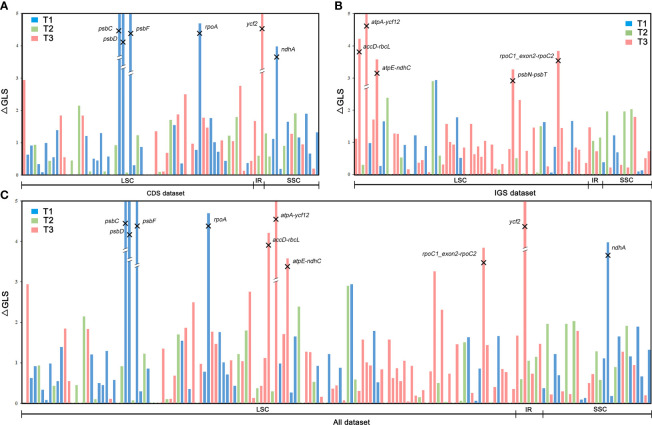
Distribution of phylogenetic signal supporting the three alternative topologies for the phylogenetic position of Dennstaedtiaceae based on gene-wise log-likelihood scores (ΔGLS) across the **(A)** CDS, **(B)** IGS, and **(C)** All datasets.

Although both biological and analytical factors influence phylogenetic inference (Bower, 1926; Mickel, 1973; Smith, 2012), the first step to understanding why different phylogenomic data matrices (or different analyses of the same data matrix) yield contradictory topologies is the precise quantification of the phylogenetic signal and identification of the genes or sites that gave rise to such conflict (Shen et al., 2017). The phylogenetic signal within the three sets of conflicting topologies (T1, T2, and T3) of Dennstaedtiaceae (**Figure 3**) across the 30 datasets was evaluated following the methods by Smith (2012), Shen et al. (2017), Gonçalves et al. (2019), Walker et al. (2019) and Zhang et al. (2020a). We first calculated the site-wise log-likelihood scores (SLS) for T1, T2 and T3. Next, we calculated the difference in site-wise log-likelihood scores (ΔSLS) among T1, T2 and T3 for every site in a given dataset. By summing the ΔSLS scores of all sites for every gene in a given dataset, we then obtained the difference in gene-wise log-likelihood scores (ΔGLS) among T1, T2 and T3. These calculations were all based on the concatenation data matrix and the same models using RAxML v8.2.12 (option -f G). Generally, tiny subsets of large data matrices, especially genes with abnormal phylogenetic signals, may also drive the resolution of specific nodes and influence phylogenetic inference (Shen et al., 2017). Therefore, to reduce the conflict at the positioning of Dennstaedtiaceae in the three topologies (**Figure 3**), the abnormal loci according to the phylogenetic signal analysis were identified and removed (Shen et al., 2017; Walker et al., 2019; Zhang et al., 2020a). The average ΔGLS was calculated for each gene in the CDS, IGS and All datasets, and the standard deviation was used to identify the outliers; loci with the average ΔGLS value greater than the upper bound or smaller than the lower bound of a Gaussian-like distribution were defined as the outlier loci. The lower and upper bound were determined as follows:


$$\text{Upper bound }=\text{ min(max(x), μ }+\;3^{*}\text{σ})$$

$$\text{Lower bound }=\text{ max(min(x), μ }+\;3^{*}\text{σ})$$

where max(*x*), min(*x*), μ, and σ indicate the maximum, minimum, average, and standard deviation, respectively, for a set of ΔGLS values (Shen et al., 2017). Subsequently, six outlier loci were removed from the CDS dataset to generate the “CDS-no-outlier” dataset (**Figure 5**), five from the IGS dataset to generate the “IGS-no-outlier” dataset (**Figure 5**) and ten from the All dataset to generate the “All-no-outlier” dataset (**Figure 5**). Phylogenetic trees were then reconstructed using IQ-TREE and ASTRAL as described previously, and phylogenetic signal was recalculated to assess the effect of loci removal.

### Hypothesis test for topologies

The approximately unbiased (AU) test (Shimodaira, 2002), Kishino–Hasegawa (KH) test (Kishino and Hasegawa, 1989), Shimodaira–Hasegawa (SH) test (Shimodaira and Hasegawa, 1999; Goldman et al., 2000), and weighted Shimodaira–Hasegawa (WSH) test (Shimodaira, 1993; Shimodaira, 1998; Shimodaira and Hasegawa, 1999; Buckley et al., 2001) implemented in CONSEL v1.20 (Hidetoshi and Masami, 2001) were applied to each dataset to test which topology was statistically better among the three alternative topologies (**Figure 3**) for all the datasets. These tests were conducted using the multi-scale bootstrap technique based on the site-wise log-likelihood scores, calculated in RAxML (option -f G).

## Results

### Characteristics of Dennstaedtiaceae plastomes

**Figure 6:**
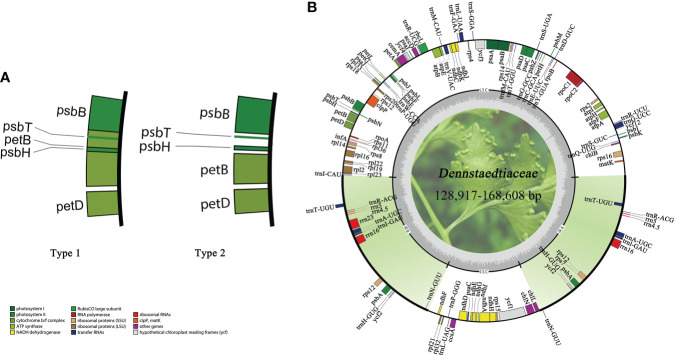
Characteristics of Dennstaedtiaceae plastomes. **(A)** A 4kb inversion observed in some plastomes, which was defined as type 1. **(B)** Plastid genome map of Dennstaedtiaceae. Genes drawn inside the circle are transcribed clockwise, whereas those outside the circle are transcribed counterclockwise.

All plastid genomes were successfully assembled and annotated. The plastomes of Dennstaedtiaceae species differed in sequence length (**Supplementary Table S1**). The maximum difference in overall sequence length of 39,691 bp was observed between *Dennstaedtia spinosa* (168,608 bp) and *Dennstaedtia producta* (128,917 bp). All Dennstaedtiaceae possessed the typical quadripartite structure of most fern plastomes with each region occupying a similar percentage of the plastome in the different species (LSC 48.7%–57.6%, IR 19.2%–15.0%, SSC 7.8%–15.0%) and a GC content approximately 41.5%–45.5%. Besides, a 4 kb inversion was found in the LSC region of some plastomes (**Supplementary Table S1**), and this phenomenon of inversion was defined as type 1 (**Figure 6**).

### Phylogenetic relationships among major lineages of Dennstaedtiaceae

**Figure 7:**
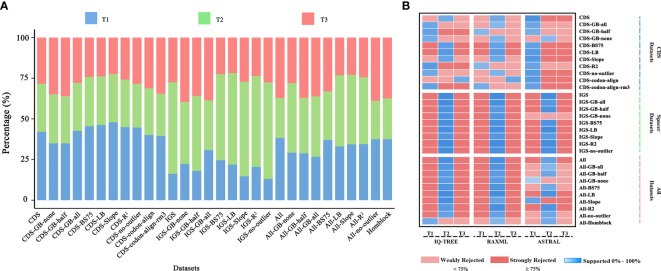
**(A)** Percentage of loci supporting each of the three alternative topological hypotheses across the 30 datasets, based on gene-wise likelihood scores; **(B)** Meta-analysis of species trees. Blue indicates the topology inferred from the dataset, and shades show the level of ultrafast bootstrap (UFBoot) values of IQ-TREE (0%–100%), rapid bootstrap (RBS) values of RAxML (0%–100%), or local posterior probability (LPP) values of ASTRAL (0–1.0). Red indicates rejection of the topology. A standard 75% of UFBoot/RBS or 0.75 of LPP were used for strong rejection.

**Figure 8:**
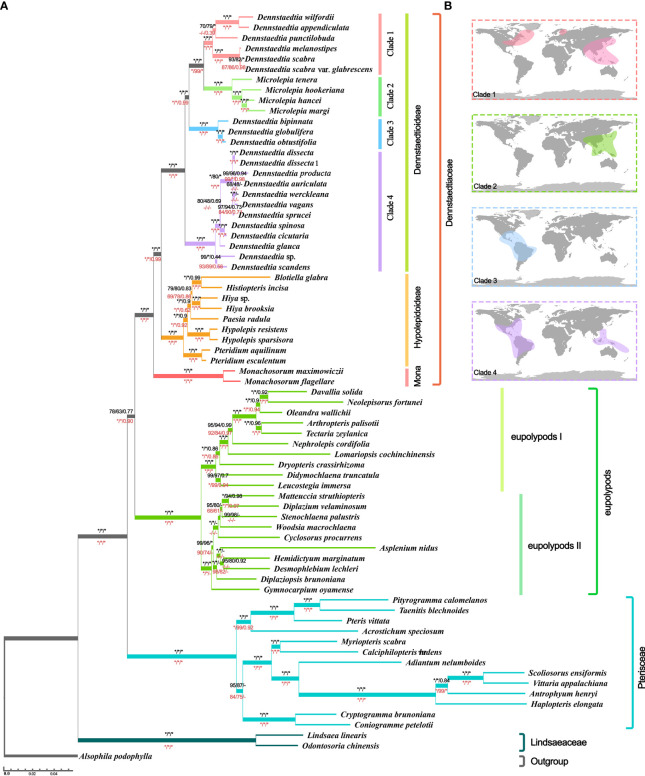
Species tree **(A)** Tree topology and branch lengths obtained from the IQ-TREE based on “CDS-codon-align-rm3” matrix. Numbers at the nodes represented the ultrafast bootstrap (UFBoot) values of IQ-TREE, rapid bootstrap (RBS) values of RAxML, and local posterior probability (LPP) values of ASTRAL. The black values above the branches obtained from “CDS-codon-align-rm3” matrices and red values below the branches obtained from amino acid matrices (**Figure S2**). Moreover, the asterisks (*) indicated 100% UFBoot/ RBS or 1.0 LPP, the hyphen **(-)** indicated support absent from the corresponding tree. **(B)** Global distribution of Dennstaedtioideae (Dennstaedtioid clade) species in clades1-4 obtained from the Global Biodiversity Information Facility.

Even though the phylogenetic relationship between Dennstaedtiaceae, Pteridineae and eupolypods based on the plastid genome showed conflicts in different regions, the present study’s analyses significantly clarified the main relationship between them. Comparing the results from the 30 datasets (**Figures 7**, **8**, **S2**; **Table 1**; **Supplementary_trees_file**), the “CDS-codon-align-rm3” dataset consistently supported the T1 topology in all analyses (ML trees, MSC tree, phylogenetic signals and five topology testing) and with highest log-likelihood value (-488333.170132 in RAxML, -488562.454 in IQ-TREE; **Table S3**) among all consensus datasets. Meanwhile, it was also similar to the topological structure of the amino-acid sequence (**Figures 8**, **S2**). In summary, the topology of the “CDS-codon-align-rm3” dataset inferred by ML was selected as our main reference or summary tree, and to infer the phylogenetic relationship within the base of polypod ferns. The species trees of “CDS-codon-align-rm3” dataset (**Figure 8**; UFBoot = 78% (IQ-TREE); RBS = 60% (RAxML); LPP = 0.78 (ASTRAL), the same below) and the amino acid dataset (UFBoot = 100%; RBS = 100%; LPP = 0.93; **Figure S2**) all revealed that Dennstaedtiaceae and eupolypods are sister clades, and together sister to Pteridineae.

**Table 1:** Statistical tests of alternative hypotheses on the phylogenetic relationships of Dennstaedtiaceae.

Database	Support topology of species tree	*P* value of T1					*P* value of T2					*P* value of T3				
		AU	KH	SH	WKH	WSH	AU	KH	SH	WKH	WSH	AU	KH	SH	WKH	WSH
CDS	–	0.5140	**0.5050**	**0.6540**	**0.5050**	**0.6550**	**0.5490**	0.4950	0.6470	0.4950	0.6470	0.0370	0.0640	0.1160	0.0620	0.1150
CDS-GB-all	T1	**0.6590**	**0.6320**	**0.7750**	**0.6320**	**0.7660**	0.4280	0.3680	0.5050	0.3680	0.5210	0.0590	0.0770	0.1340	0.0770	0.1420
CDS-GB-half	T1	**0.7180**	**0.6630**	**0.8000**	**0.6630**	**0.7880**	0.3650	0.3370	0.4640	0.3370	0.4840	0.0450	0.0670	0.1110	0.0670	0.1230
CDS-GB-none	T1	**0.5980**	**0.5650**	**0.7190**	**0.5650**	**0.7160**	0.5320	0.4350	0.5910	0.4350	0.5960	0.1660	0.1840	0.3050	0.1840	0.3110
CDS-BS75	T2	0.4080	0.3580	0.4910	0.3580	0.5060	**0.6750**	**0.6420**	**0.7820**	**0.6420**	**0.7710**	0.0440	0.0690	0.1150	0.0690	0.1240
CDS-LB	T2	0.2880	0.2740	0.3740	0.2740	0.4080	**0.7520**	**0.7260**	**0.8460**	**0.7260**	**0.8320**	0.0110	0.0250	0.0380	0.0250	0.0480
CDS-Slope	T2	0.4790	0.4320	0.5770	0.4320	0.5850	**0.5720**	**0.5680**	**0.7120**	**0.5680**	**0.7080**	0.0140	0.0420	0.0730	0.0420	0.0780
CDS-R2	T1	**0.7330**	**0.6730**	**0.8130**	**0.6730**	**0.8000**	0.3330	0.3270	0.4450	0.3270	0.4710	0.0410	0.0530	0.0930	0.0530	0.1010
CDS-no-outlier	T1	**0.6700**	**0.6400**	**0.7860**	**0.6400**	**0.7740**	0.3750	0.3600	0.4830	0.3600	0.5000	0.0160	0.0300	0.0560	0.0300	0.0600
CDS-codon-align	T3	0.4520	0.3980	0.5510	0.3980	0.5550	0.2710	0.2740	0.4100	0.2740	0.4160	0.6600	**0.6020**	**0.7570**	**0.6020**	**0.7560**
CDS-codon-align-rm3	T1	**0.6770**	**0.6360**	**0.7770**	**0.6360**	**0.7690**	0.4230	0.3640	0.5000	0.3640	0.5150	0.0790	0.0930	0.1510	0.0930	0.1650
IGS	T2	0.0040	0.0040	0.0040	0.0040	0.0060	**0.9970**	**0.9960**	**0.9980**	**0.9960**	**0.9980**	0.0001	0.0010	0.0010	0.0010	0.0010
IGS-GB-all	T2	0.0200	0.0240	0.0240	0.0240	0.0410	**0.9770**	**0.9600**	**0.9820**	**0.9600**	**0.9790**	0.0440	0.0400	0.0410	0.0400	0.0720
IGS-GB-half	T2	0.0170	0.0230	0.0250	0.0230	0.0440	**0.9180**	**0.9030**	**0.9510**	**0.9030**	**0.9400**	0.1110	0.0970	0.1050	0.0970	0.1640
IGS-GB-none	T2	0.0870	0.0870	0.0910	0.0870	0.1600	**0.9190**	**0.8900**	**0.9250**	**0.8900**	**0.9210**	0.1310	0.1100	0.1130	0.1100	0.1940
IGS-BS75	T2	0.0610	0.0610	0.0650	0.0610	0.1060	**0.9500**	**0.9390**	**0.9760**	**0.9390**	**0.9700**	0.0030	0.0060	0.0060	0.0060	0.0100
IGS-LB	T2	0.0020	0.0030	0.0030	0.0030	0.0040	**0.9990**	**0.9970**	**0.9990**	**0.9970**	**0.9990**	0.2470	0.2140	0.2250	0.2140	0.3730
IGS-Slope	T2	0.0001	0.0002	0.0002	0.0002	0.0002	**0.8180**	**0.7860**	**0.8260**	**0.7860**	**0.8230**	0.2090	0.1970	0.2120	0.1970	0.3520
IGS-R2	T2	0.0080	0.0090	0.0090	0.0090	0.0150	**0.9950**	**0.9910**	**0.9960**	**0.9910**	**0.9960**	0.0060	0.0060	0.0060	0.0060	0.0100
IGS-no-outlier	T2	0.0430	0.0400	0.0430	0.0400	0.0720	**0.9710**	**0.9600**	**0.9830**	**0.9600**	**0.9810**	0.0060	0.0080	0.0090	0.0080	0.0170
All	T2	0.1040	0.0960	0.1210	0.0960	0.1630	**0.8970**	**0.9040**	**0.9640**	**0.9040**	**0.9530**	0.0004	0.0010	0.0010	0.0010	0.0010
All-GB-all	T2	0.2040	0.1850	0.2560	0.1850	0.2980	**0.8280**	**0.8150**	**0.9040**	**0.8150**	**0.8960**	0.0180	0.0190	0.0260	0.0190	0.0370
All-GB-half	T2	0.3130	0.2790	0.3890	0.2790	0.4150	**0.7570**	**0.7210**	**0.8420**	**0.7210**	**0.8340**	0.0350	0.0460	0.0740	0.0460	0.0880
All-GB-none	T2	0.3420	0.2810	0.4030	0.2810	0.4290	**0.7650**	**0.7190**	**0.8340**	**0.7190**	**0.8290**	0.1110	0.1070	0.1700	0.1070	0.1950
All-BS75	T2	0.1920	0.2130	0.2840	0.2130	0.3270	**0.8120**	**0.7870**	**0.8950**	**0.7870**	**0.8780**	0.0010	0.0030	0.0030	0.0030	0.0050
All-LB	T2	0.0220	0.0250	0.0260	0.0250	0.0440	**0.9780**	**0.9750**	**0.9930**	**0.9750**	**0.9890**	0.0000	0.0000	0.0000	0.0000	0.0000
All-Slope	T2	0.3740	0.3500	0.4840	0.3500	0.5050	**0.6710**	**0.6500**	**0.7820**	**0.6500**	**0.7730**	0.0210	0.0350	0.0560	0.0350	0.0670
All-R2	T2	0.0950	0.1050	0.1330	0.1050	0.1810	**0.9130**	**0.8950**	**0.9550**	**0.8950**	**0.9480**	0.0010	0.0030	0.0040	0.0030	0.0060
All-no-outlier	T2	0.3700	0.3570	0.4840	0.3570	0.5040	**0.6370**	**0.6430**	**0.7790**	**0.6430**	**0.7680**	0.0010	0.0040	0.0060	0.0040	0.0070
All-Homblock	T1	**0.3890**	**0.3500**	**0.4780**	**0.3500**	**0.5000**	0.6740	0.6500	0.7840	0.6500	0.7760	0.0410	0.0590	0.0950	0.0590	0.1090

*P*< 0.05 refuted monophyly; *P* > 0.05 do not refute the possibility of monophyly. The en dash (–) indicated that the result supported more than one topology. *P* value in Bold indicated the topology supported by the five topological hypotheses testing.

The three clades (Dennstaedtioideae, Hypolepidoid clade and Monachosoroideae) of Dennstaedtiaceae were all confirmed as monophyletic with strong support (UFBoot = 100%; RBS = 100%; LPP = 1.0) in all analyses (**Figure 8**; **Supplementary_trees_file**), and the systematic relationships of most genera were also relatively clear, except *Paesia*. Across all datasets, 64.51% of IQ-TREE, 48.39% of RAxML, and 74.19% of ASTRAL results supported *Paesia* as sister group of *Blotiella*, *Histiopteris*, and *Hiya*. Only 29.03% of IQ-TREE, 45.16% of RAxML and 19.35% of ASTRAL results supported *Paesia* as sister to *Blotiella* and *Histiopteris*; and 6.45% of IQ-TREE, 6.45% of RAxML and 6.45% of ASTRAL results supported *Paesia* as sister to *Hiya* (**Supplementary_trees_file**). It is worth noting that the species trees inferred by CDS and CDS-derived datasets all supported the (((*Blotiella*, *Histiopteris*), *Hiya*), *Paesia*) topology, except for “CDS-GB-None” dataset inferred by ASTRAL. Besides, *Dennstaedtia* is paraphyletic, divided into three branches and with *Microlepia* embedded, with strong support (UFBoot = 100%; RBS = 100%; LPP = 1.0; **Figure 8**; **Supplementary_trees_file**). According to the information data of Global Biodiversity Information Facility (GBIF.org, 2021), clade 1 includes species distributed in East Asia-North America, clade 3 included species distributed in central and southern America, and clade 4 included species distributed in tropical America and Southeast Asia (**Figure 8**). Plastome linearized maps of all samples showed that clade 1 had syntenic blocks, which were absent in other clades; the species of clades 1-3 also had another common syntenic blocks, which were not found in clade 4 (**Figure S3**).

### Conflicting phylogenetic signal in the plastome

Phylogenetic analyses of 30 datasets obtained from CDS and intergenic spacer regions yielded 2,642 trees, consisting of 2,552 gene trees inferred for each dataset plus 90 species trees inferred using different tree search methods (**Supplementary_trees_file**). Conflicting topologies depicting the relationships between Dennstaedtiaceae, Pteridineae and eupolypods were obtained from the different datasets despite the multiple strategies used to reduce systematic error (**Figure 7**). The ML analysis of the CDS and CDS-derived datasets resulted in conflicting topologies depending on the dataset and on the method (IQ-TREE vs RAxML). For example, IQ-TREE supported T1 topology in “CDS-GB-all” matrix, while RAxML supported T2 topology (**Figure 7**). However, the MSC method, which deals with heterogeneity among gene trees, demonstrated consistent relationships and mainly supported T1 ((eupolypods, Dennstaedtiaceae),Pteridineae). The different strategies and methods of analysis of the IGS and IGS-derived datasets, excluding the “IGS-GB-half” dataset, consistently supported the T2 topology ((Dennstaedtiaceae,Pteridineae),eupolypods). Meanwhile, for All and All-derived datasets, the majority of the phylogenetic results also supported the T2 topology (**Figure 7**).

Furthermore, the phylogenetic signal supporting these conflicts was quantified and the proportions of genes supporting the alternative topologies were visualized for each dataset (**Figure 7**; **Supplementary Table S4**). All the 166 loci with strong signals favoring T1, T2 or T3 were unevenly distributed in the different plastome regions. Further examination of the ΔGLS values for T1, T2 and T3 (**Figure 3**) in 11 CDS/CDS-derived datasets revealed that T1 had a higher proportion of supporting genes (10/11; 34.6%–47.2%) than those favoring either T2 (0/11; 25.9%–32.4%) or T3 (2/11; 22.2%–35.8%). Meanwhile, T2 (7/9; 30.2%–59.2%) had a higher proportion of supporting genes than those favoring either T1 (0/9; 13.2%–31.8%) or T3 (2/9; 21.5%–40.3%) in nine IGS/IGS-derived datasets. The results from the All/All-derived datasets were similar to those obtained from the IGS/IGS-derived datasets, with T2 (7/10; 25.0%–44.1%) having a higher proportion of supporting genes than those favoring either T1 (2/10; 26.3%–44.6%) or T3 (2/10; 22.7%–37.5%). A summary of the phylogenetic signal of the genes is presented in **Supplementary Tables S4**, **S5**.

The support for the alternative topologies (**Figure 3**) was further assessed *via* KH-, SH-, WSH-, WKH-, and AU-tests (**Table 1**). **Table 1** shows the different datasets of the plastomes that support different hypotheses. Among them, 54.5% of the CDS/CDS-derived datasets supported the T1 hypothesis, suggesting that Dennstaedtiaceae and eupolypods were sister groups. Meanwhile, 27.3% supported T2, and only 9.0% supported T3. IGS/IGS-derived and All/All-derived datasets mainly supported the T2 hypothesis.

## Discussion

### Deep phylogenetic relationships of Dennstaedtiaceae

The results of different species tree inference methods in 30 datasets showed that the phylogeny within the Dennstaedtiaceae was relatively stable (**Figure 8**; **Supplementary_trees_file**). The three major clades of Dennstaedtiaceae correspond to the three subfamilies, Dennstaedtioideae (Dennstaedtioid clade), Hypolepidoideae (Hypolepidoid clade) and Monachosoroideae (**Figure 8**), consistent with a recently reported phylogeny (Schwartsburd et al., 2020). It also supported the conclusion that Monachosoroideae was the earliest divergent branch of Dennstaedtiaceae (Liu et al., 2008; Rothfels et al., 2015; Schwartsburd et al., 2020). Besides, monophyly was well supported for the genera of the Hypolepidoid clade (*Hypolepis*, *Pteridium*, *Blotiella*, *Histiopteris*, *Paesia*, *Hiya*) and Monachosoroideae (*Monachosorum*) in all datasets (Rothfels et al., 2015; Liu, 2016; Schwartsburd et al., 2020). In our plastid phylogeny, *Pteridium* was recovered as sister to the remaining Hypolepidoid clade species and *Paesia* was sister to the *Blotiella*, *Histiopteris*, and *Hiya*, contrary to what was found by Schwartsburd et al. (Schwartsburd et al., 2020). The relationship among Hypolepidoid clade genera need further study with comprehensive taxonomic sampling and integrative evidences.

The monophyly of Dennstaedtioideae genera, especially *Dennstaedtia*, remained uncertain (Perrie et al., 2015; PPGI, 2016; Shang et al., 2018; Schwartsburd et al., 2020). In our analysis, *Dennstaedtia* was identified as paraphyletic and divided into three branches, as previously shown (Perrie et al., 2015; Shang et al., 2018; Schwartsburd et al., 2020; Wang et al., 2021). In the characteristics of plastomes, a large 4 kb inversion (*petB*-*psbH*) in the LSC region (type 1; **Figure 6**) was mainly distributed in clade 1, clade 2, clade3 (except for *Microlepia marginata*) and *Blotiella glabra* (**Figure 8**). This inversion was not found in other lineages of Dennstaedtiaceae, probably related to their plastid structural characteristics. The analysis of genetic structure (**Figure 6**), plastome linearized maps (**Figure S3**) and geographical distribution (**Figure 8**) in the present study revealed that the species of clades 1-4 have unique characteristics. Besides, according to the particular phylogenetic position of *Microlepia* and the distinguishing characters between *Microlepia* and *Dennstaedtia* s.l. (e.g., sori position, indusium shape, spore ornamentation and the connection of grooves between rachis and pinna rachis; Wang et al., 2021), we support the segregation of subfamily Dennstaedtioideae into smaller genera, including *Dennstaedtia* s.s. (clade 4), *Microlepia* (clade 2), *Sitobolium* Desvaux (clade1) in East Asia-North America clade, and a new genus in tropical America clade (clade 3). This treatment is consistent with the proposal of conservation of *Dennstaedtia* with *D. dissecta* as type published on TAXON (Triana-Moreno et al., 2022). If the *Dennstaedtia* s.l. is segregated into multiple genera, it means the new type species of *Dennstaedtia* should be accepted. Apart from the results mentioned above and plant size, we have not yet found any convincing synapomorphies within clades 1, 2 and 4. Further taxonomic research is needed to clearly understand the division.

### Relationships within the early branches of polypod ferns

Polypods include more than 82% of extant ferns, and enormous progress has been made in clarifying their phylogenetic relationships using plastid genomics and transcriptomics (Lu et al., 2015; Perrie et al., 2015; Rothfels et al., 2015; PPGI, 2016; Qi et al., 2018; Shang et al., 2018; Shen et al., 2018; Du et al., 2021). Our results (**Figure 8**) showed that five major lineages (eupolypods I, eupolypods II, dennstaedtioids, pteroids, lindsaeoids) were recovered in agreement with the consensus hypothesis (Lu et al., 2015; Perrie et al., 2015; Rothfels et al., 2015; PPGI, 2016; Qi et al., 2018; Shang et al., 2018; Shen et al., 2018; Du et al., 2021), while the position of Dennstaedtiaceae was different from the previous studies using plastid genes (Schuettpelz and Pryer, 2007; Kuo et al., 2011; Testo and Sundue, 2016), and even plastid genomes (Du et al., 2021). Studies have inferred different topologies using different plastid genes and strategies, indicating plastid phylogenomic conflict as a significant obstacle to the understanding of relationships of these taxa (Gonçalves et al., 2019; Walker et al., 2019; Zhang et al., 2020a).

Even though it is currently believed that the main cause of mass conflicts in phylogeny are stochastic and systematic errors, or misspecifications of the evolutionary models (Walker et al., 2019; Daniell et al., 2021), we applied multiple strategies (e.g., removal of ambiguously aligned regions, high long-branch genes, low BS genes, and outlier genes) to minimize systematic and phylogenetic analysis errors, and still clearly observe the existence of plastid conflicts in phylogenetic inference (**Figure 7**). Only comparing the results of the various inferred trees does not solve the problem of choosing the best species tree. Using the method of phylogenetic signals and topologies hypothesis testing can help us clearly quantify the conflict situations within the phylogenetic tree. After comparing the results inferred by different methods among all datasets, we found that “CDS-codon-align-rm3” matrix consistently supported the T1 topology in all analysis, and was similar to the topological structure of the amino-acid sequence (**Figure 8**, **S2**). Besides, the phylogenetic trees constructed by Rothfels et al. (2015), Shen et al. (2018), Qi et al. (2018) and One Thousand Plant Transcriptomes Initiative (2019) based on 25, 1334/2391, 533 and 410 low- or single-copy nuclear genes, respectively, also supported the T1 topology. The morphological characters (e.g., indusium, sporangium and spore shape) are consistent with our result (**Figures 8**, **S2**). First, the unstable structure of the spherical sporangia in Pteridaceae, including the variable annulus and short sporangial stalk, indicates that these characters of the sporangia are relatively original and close to those with an oblique annulus in early leptosporangiates (Bower, 1926; Shen et al., 2018). Second, Dennstaeditaceae with two indusial is more related to eupolypod ferns (Mickel, 1973; Shen et al., 2018), rather than Pteridaceae with one false indusium. Finally, the spore shape of most Pteridaceae species are trilete (Zhang et al., 2013), while Dennstaetiaceae displays two spore shapes (Yan et al., 2013; Shang et al., 2018), which evolved from trilete (Monachosoroideae, Dennstaedtioideae and *Pteridium*) to monolete spores (*Blotiella*–*Hypolepis*), and are more closely related to eupolypod ferns with monolete spores (http://www.mobot.org/MOBOT/Research/APweb/ ).

### Conflicting topologies inferred from plastomes

Our current knowledge of land plant relationships is mainly based on concatenated plastid markers and ML inference (Barrett et al., 2013; Lu et al., 2015; Du et al., 2021; Guo et al., 2021). This approach has been justified by the assumption that plastid genes are inherited as a single coalescent gene (c-gene) and that the individual genes produce congruent trees (Gonçalves et al., 2019). However, some researchers found that different plastid genes or sequence types (coding vs. non-coding) provide conflicting resolutions at some key nodes in angiosperms, such as legumes (Zhang et al., 2020a) and Zygophyllales (Gonçalves et al., 2019). The present study detected a similar situation in ferns (**Figure 7**; **Table 1**), indicating that heterogeneity among plastid genes is a common phenomenon in vascular plants. If the viewpoint that plastome should not be treat as c-genes is correct (Gonçalves et al., 2019; Walker et al., 2019), combining various plastid genes into a single analysis may conflate multiple phylogenetic signals, muddying the overall inference of both topology and branch lengths, and challenging downstream divergence times, diversification and character evolution analyses (Daniell et al., 2021). In fact, the question of whether the plastome is the c-gene or m-gene actually needs more research to confirm (Doyle, 2022), but it is undeniable that examine plastid conflicts in detail when used to inferred phylogenetic tree is important.

Various strategies (e.g., removal of uninformative regions, low BS genes, outlier genes and saturated genes) and inference methods (**Figure 4**, **Figure 5**, **Figure 7**, **Figure S1**) were used to avoid systematic error; however, this approach removed only a part of the conflicting information (Zhang et al., 2020a). Most conflicts remained unresolved, which may be related to the characteristics of plastid genes, such as evolutionary rate (Zhang et al., 2020b; Vankan et al., 2022) and GC content (Smith, 2012). Besides, other biological factors, such as heteroplasmic recombination, may also have led to plastid gene heterogeneity. The plastome is usually considered uniparentally inherited and free of sexual recombination; however, it has been shown to undergo inter-plastome recombination in multiple studies (Sullivan et al., 2017; Sancho et al., 2018). Although both biological and analytical factors influence phylogenetic inference, the precise quantification of the phylogenetic signals of each plastid locus (**Figure 5**; **Supplementary Table S5**) and identification of the loci that give rise to conflicts (Shen et al., 2017), may help us understand the actual evolutionary pattern.

Synthesizing the study results, we found that using Gblocks to exclude ambiguously aligned regions with relaxed, default and strict parameters, removing low BS or outlier genes produced no more conflict reduction than excluded loci with high levels of excessive substitutional saturation. Besides, according to the phylogenetic signals results (**Figure 7**), we found that CDS/CDS-derived datasets mainly support T1 topology, while the results from the ML method yielded three topologies. This phenomenon implies that quantify phylogenetic signal is necessary when determining phylogenetic relationships. Interestingly, the results of the MSC method used to reduce the effect of genetic heterogeneity (Edwards and Batley, 2010; Zhang et al., 2021) were consistent with the T1 species tree (except for the CDS-GB-None dataset; **Figure 8**). Consistent with Walker et al. (Walker et al., 2019), MSC was more consistent than ML when considering variation or inconsistencies in phylogenetic signal across plastid genes (Chou et al., 2015). Contrary to the CDS datasets (**Figure 7**), IGS/IGS-derived datasets mainly supported T2. The intergenic spacers are non-functional regions with a faster rate of evolution (Kress et al., 2005; Smith, 2012; Amiryousefi et al., 2018; Trujillo-Argueta et al., 2021); therefore, they probably get easily saturated and lose phylogenetic information, leading to trees inconsistent with the clade history (Xia et al., 2003). Thus, when performing phylogenetic reconstruction at higher taxonomic levels, researchers chose to use the coding regions (Li et al., 2019a; Li et al., 2019b; Du et al., 2021) or slowly evolving plastid genes (Jian et al., 2008; Regier et al., 2008).

### Application and implication of plastid phylogenomic

At present, public databases, such as NCBI, China National Gene Bank (CNGB; https://www.cngb.org/ ), Chloroplast Genome Database (ChloroplastDB; http://chloroplast.cbio.psu.edu/ ), have accumulated plastomes of more than 10,000 species (**Figure 1**), improving taxonomic coverage in studies. Some studies have shown that taxa coverage has a specific influence on the stability of phylogeny (Heath et al., 2008; Nabhan and Sarkar, 2012), and when taxon sampling is limited, phylogeny may be biased towards some wrong topological structure (Aznar-Cormano et al., 2015). Therefore, public databases should be used efficiently to expand the sampling of target groups for solving complex group relations.

Nucleic acid sequences have become the most predominant component in phylogeny (Brown, 2002), mainly because DNA yields more phylogenetic information than protein due to the degeneracy of the genetic code (Gupta, 1998; Brown, 2002). However, compared with protein sequences, the nucleotide sequences often show substitution saturation due to more mutation sites (Page and Holmes, 1998; Nei and Kumar, 2000) and lose phylogenetic information more quickly, leading to wrong trees (Xia et al., 2003). In particular, the bases at the third codon positions in distantly related taxa that diverged from each other a long time ago may have changed several times, so that the actual bases found at these positions are random and their information content is virtually nil (Gupta, 1998). For phylogenetic analyses involving distantly related taxa, the increased information content in nucleic acid sequences may be an illusion and, in most cases, a major liability, as it may reduce the signal to noise ratio in the dataset (Gupta, 1998; Jian et al., 2008). This is illustrated by the fact that many studies have found that topologies inferred from nucleotide sequences were inconsistent with those inferred from amino acid sequences (Gonçalves et al., 2019; Zhang et al., 2020a).

In this study, the saturated genes or the fast-evolving sites were removed to generate some of the datasets, namely “CDS-*R^2^*/slope” and “CDS-codon-align-rm3” (**Figure S1**). When the plastid conflicts are very widespread, removing the saturated genes (i.e., those with a higher *R^2^* value) or the third codon positions (i.e. those with a faster evolution rate) will reduce the conflicts in species tree inference, and produced a topological structure consistent with the one obtained from amino acid sequences (**Figure 6**, **Figure 7**, **S2**; **Table 1**). Therefore, genes with conservative evolution rate should be selected to reduce internal conflicts in plant phylogenetics, especially in ferns, an ancient lineage of more than 400 million years of independent evolution (Sessa et al., 2014; Rothfels et al., 2015; Qi et al., 2018; Shen et al., 2018). Besides, the species trees inferred from the nucleotide and amino acid sequences should be compared to determine the systematic relationship of distantly related taxa (**Figure 4**).

## Data availability statement

The data presented in this study are deposited in the National Center for Biotechnology Information (https://www.ncbi.nlm.nih.gov/), and the accession number(s) can be found in the article/**Supplementary Table 1**. The link to the Supplementary trees file can be found here: https://github.com/TingWang-93/ferns.

## Author contributions

Y-HY and J-YX conceived the study. Y-HY and Y-NM designed and carried out taxon sampling. TW, T-ZL, TY, S-SC and J-PS designed and coordinated computational analyses. TW, T-ZL, Y-HY, J-YX, K-LW and J-BC wrote and revised the manuscript. All authors contributed to the article and approved the submitted version..

## Funding

This work was funded by the Strategic Priority Research Program of the Chinese Academy of Sciences (XDA19050404) and the National Natural Science Foundation of China (31370234; 32170216).

## Acknowledgments

We thank Dr. Li-Bing Zhang (Missouri Botanical Garden) for samples of *Dennstaedtia*; Mr. Hui Shang (Shanghai Chenshan Botanical Garden) for the data of *Hiya brooksiae*, *Hypolepis resistens* and *Pteridium esculentum*. We are grateful to Dr. Rong Zhang (Kunming Institute of Botany, Chinese Academy of Sciences) for helpful of discussions of our results.

## Conflict of interest

The authors declare that the research was conducted in the absence of any commercial or financial relationships that could be construed as a potential conflict of interest.

## Publisher’s note

All claims expressed in this article are solely those of the authors and do not necessarily represent those of their affiliated organizations, or those of the publisher, the editors and the reviewers. Any product that may be evaluated in this article, or claim that may be made by its manufacturer, is not guaranteed or endorsed by the publisher.

## Supplementary material

Supplementary Table 1 List of 72 studied taxa including voucher information and GenBank accession numbers.

Supplementary Table 2 List of loci removed in all datasets.

Supplementary Table 3 Characteristics of all analyzed plastome datasets.

Supplementary Table 4 Distribution of phylogenetic signal for the three alternative topologies of showing the phylogenetic position of Dennstaedtiaceae based on gene-wise log-likelihood scores (ΔGLS) across each dataset.

Supplementary Table 5 Gene-wise phylogenetic signal for the three alternative topologies of showing the phylogenetic position of Dennstaedtiaceae based on all datasets.

Supplementary Figure 1 Density plots of long-branch score, slope values and *R^2^* values for 166 loci generated using *R*.

Supplementary Figure 2 Tree topology and branch length indicated maximum-likelihood (ML) analysis based on amino acid matrix, and branches of each family are designated in different colors.

Supplementary Figure 3 Linearized map comparison of the plastid genomes of Dennstaedtioideae (clades 1-4). Syntenic blocks are shown in different colors.
