# Supplementary figures and images for: Untying the Gordian knot of plastid phylogenomic conflict: A case from ferns
Source: Front Plant Sci. 2022 Nov 24;13:918155. doi: 10.3389/fpls.2022.918155 (PMC9730426; doi:10.3389/fpls.2022.918155)

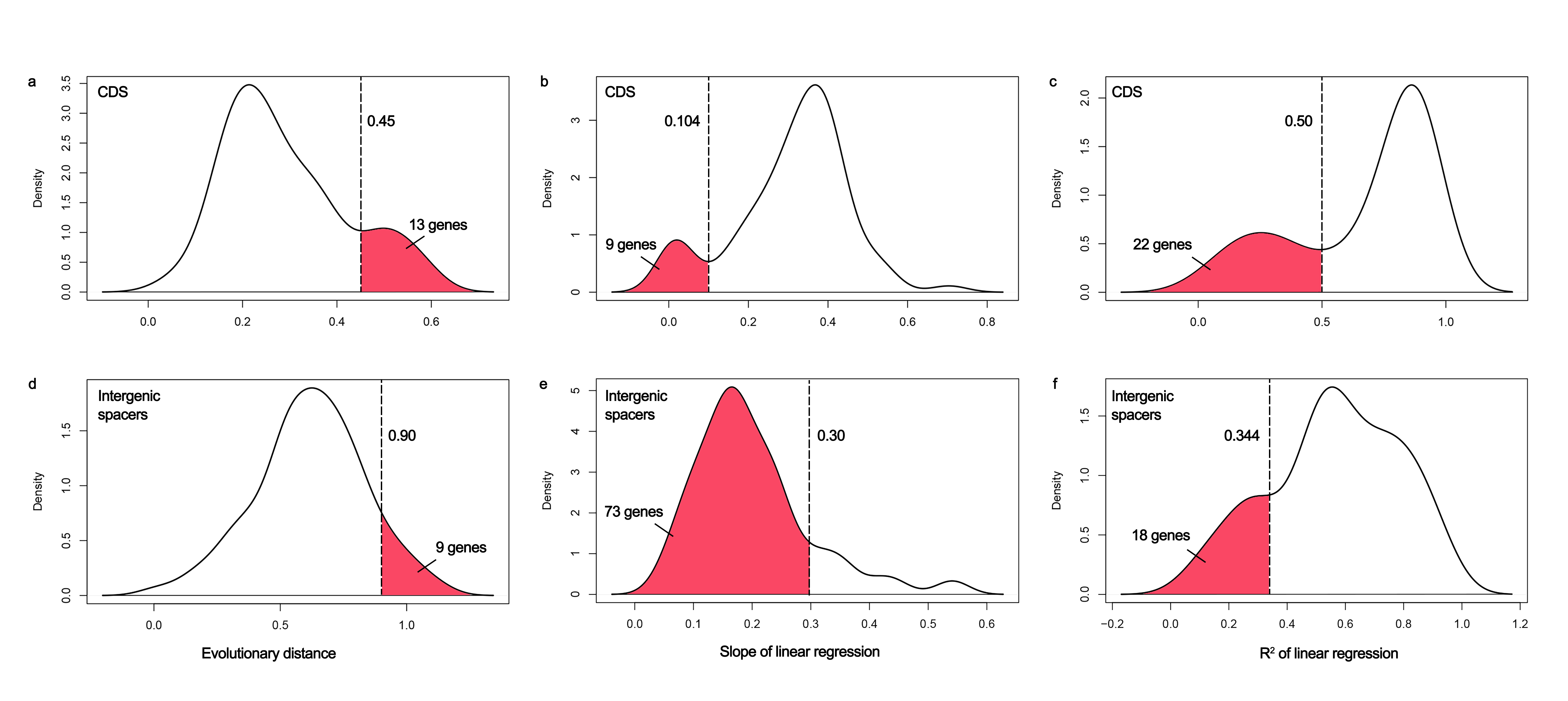

Supplement: Supplementary Figure 1 — Density plots of long-branch score, slope values and R2 values for 166 loci generated using R. [file Image_1.jpg]

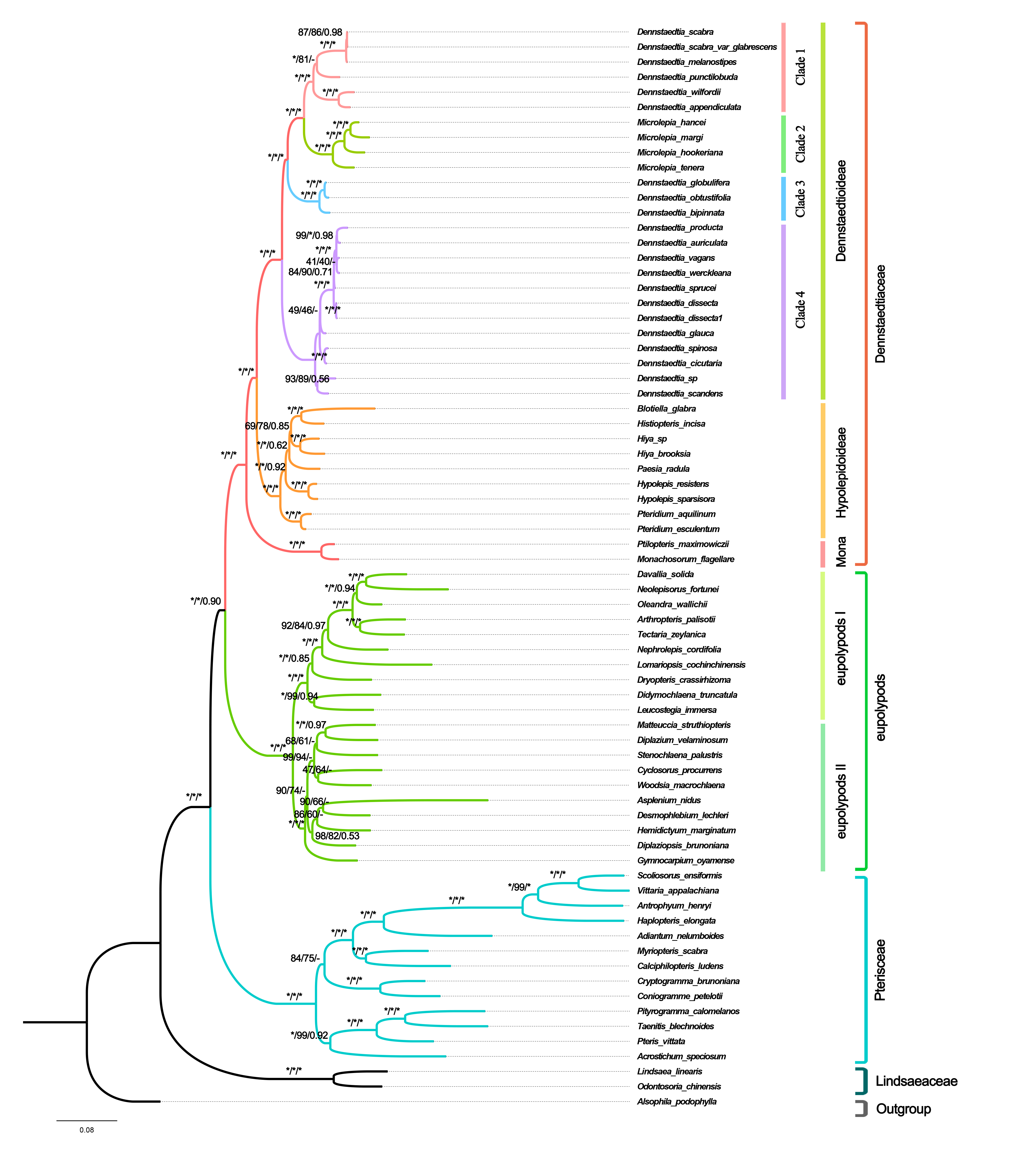

Supplement: Supplementary Figure 2 — Tree topology and branch length indicated maximum-likelihood (ML) analysis based on amino acid matrix, and branches of each family are designated in different colors. [file Image_2.jpg]

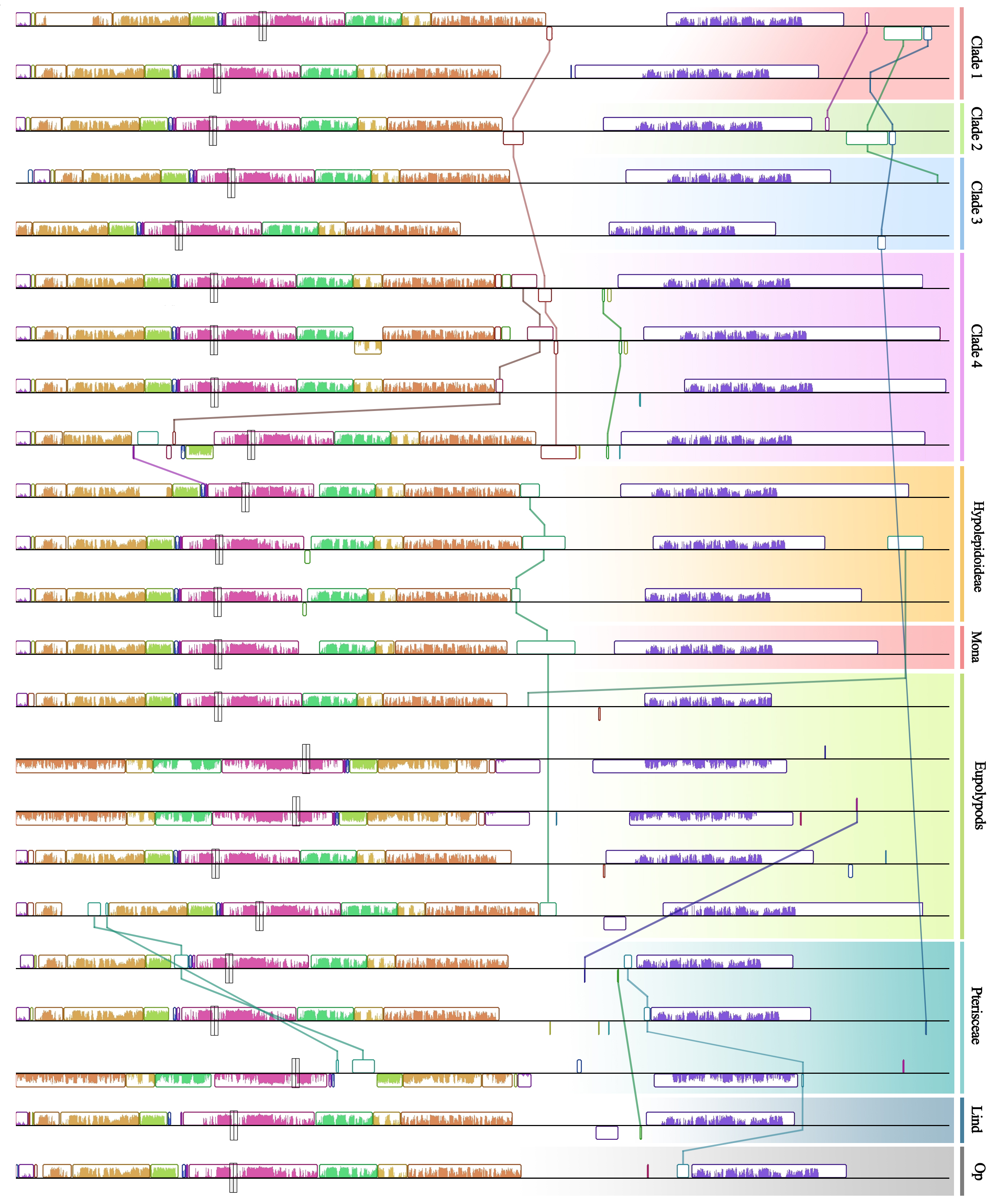

Supplement: Supplementary Figure 3 — Linearized map comparison of the plastid genomes of Dennstaedtioideae (clades 1-4). Syntenic blocks are shown in different colors. [file Image_3.jpg]
